# Validation of a multifactorial risk factor model used for predicting future caries risk with nevada adolescents

**DOI:** 10.1186/1472-6831-11-18

**Published:** 2011-05-20

**Authors:** Marcia M Ditmyer, Georgia Dounis, Katherine M Howard, Connie Mobley, David Cappelli

**Affiliations:** 1 UNLV School of Dental Medicine, 1001 Shadow Lane, MS 7425, Las Vegas, NV 89106, USA; 2 UT Health Science Center San Antonio Dental School, 7703 Floyd Curl Dr. San Antonio, TX 78229, USA

**Keywords:** dental caries, DMFT Index, Specificity, Sensitivity

## Abstract

**Background:**

The objective of this study was to measure the validity and reliability of a multifactorial Risk Factor Model developed for use in predicting future caries risk in Nevada adolescents in a public health setting.

**Methods:**

This study examined retrospective data from an oral health surveillance initiative that screened over 51,000 students 13-18 years of age, attending public/private schools in Nevada across six academic years (2002/2003-2007/2008). The Risk Factor Model included ten demographic variables: exposure to fluoridation in the municipal water supply, environmental smoke exposure, race, age, locale (metropolitan vs. rural), tobacco use, Body Mass Index, insurance status, sex, and sealant application. Multiple regression was used in a previous study to establish which significantly contributed to caries risk. Follow-up logistic regression ascertained the weight of contribution and odds ratios of the ten variables. Researchers in this study computed sensitivity, specificity, positive predictive value (PVP), negative predictive value (PVN), and prevalence across all six years of screening to assess the validity of the Risk Factor Model.

**Results:**

Subjects' overall mean caries prevalence across all six years was 66%. Average sensitivity across all six years was 79%; average specificity was 81%; average PVP was 89% and average PVN was 67%.

**Conclusions:**

Overall, the Risk Factor Model provided a relatively constant, valid measure of caries that could be used in conjunction with a comprehensive risk assessment in population-based screenings by school nurses/nurse practitioners, health educators, and physicians to guide them in assessing potential future caries risk for use in prevention and referral practices.

## Background

Although dental caries has declined significantly among school-aged children since the early 1970s, oral disease, including caries, remains a major public health challenge [1-3]. In 2004, the reported prevalence of dental caries was approximately 60% in US children ages 12 to 19, with a reported 20% in untreated tooth decay [3]. Childhood dental caries has been reported to be the most prevalent infectious disease in our nation - 5 times as common as asthma and 7 times as common as hay fever [4]. Sixty-seven percent of 12 to 17 year olds reported caries experience, with more than 7% of all children losing at least one permanent tooth to decay before reaching the age of 17 [4]. Researchers have established associations between poor oral health status and systemic diseases, genetics, behavioral, and environmental factors [5-8]. In an effort to help reduce caries prevalence, adolescents who are at greater risk can be identified through population-based screenings by school nurses/nurse practitioners, health educators, and physicians to guide them in assessing potential future caries risk so referrals can be made to dental professionals. This will also help in targeting intervention strategies, including behavior modifications [i.e., oral hygiene, dietary, fluoridation] [9]. The ability to identify these potential factors such as lifestyle, ethnicity, health status, and social conditions associated with oral health status can help classify, thus distinguish, adolescents who might be at greater caries risk. School nurses/nurse practitioners and health educators are far more likely to encounter adolescents on a regular basis than dental professionals. Therefore, it is essential that they be familiar with the various risk factors associated with dental caries to help make appropriate referral and intervention decisions.

In 2008, a study was conducted to determine the prevalence (untreated and restored lesions and untreated dental caries) and severity (DMFT Indices) among Nevada youth assessed during a statewide, school-based oral health screening initiative, while using the data to develop a theoretical caries risk screening tool for use in population-based screenings that could be validated in future studies. The original study analyzed data from a cohort of students previously screened during 2005/2006 academic school year. Inclusion criteria for participation were parental consent and student assent. The University of Nevada Las Vegas Institutional Review Board approved this initiative to assure student confidentiality.

By validating this instrument, public health practitioners could use the multifactorial Risk Factor Model with other risk assessments with confidence to guide them in prevention and referral practices. Therefore, the objective of this study was to measure the validity and reliability of this multifactorial Risk Factor Model developed for use in predicting future caries risk in Nevada adolescents in a public health setting.

## Methods

### Development of the Multifactorial Risk Factor Model

The original instrument was developed using retrospective data from a school-based, oral health screening initiative consisting of a cohort of 9,202 adolescents between ages 13-19 attending Nevada public/private high schools in the 2005/2006 academic year. Multiple regression analyses were initially used to establish the variables that significantly contributed to caries prevalence and severity (p < 0.05). Follow-up logistic regression ascertained the weight of contribution and odds ratio of the ten significant variables from the multiple regression analyses [10]. These included (in hierarchal order): 1) exposure to fluoridated water in the municipal water supply, 2) exposure to environmental smoke, 3) race, 4) age, 5) locale (metro vs. rural), 6) use of tobacco, 7) Body Mass Index (BMI), 8) dental insurance status, 9) sex, and 10) application of sealants.

**Figure 1:**
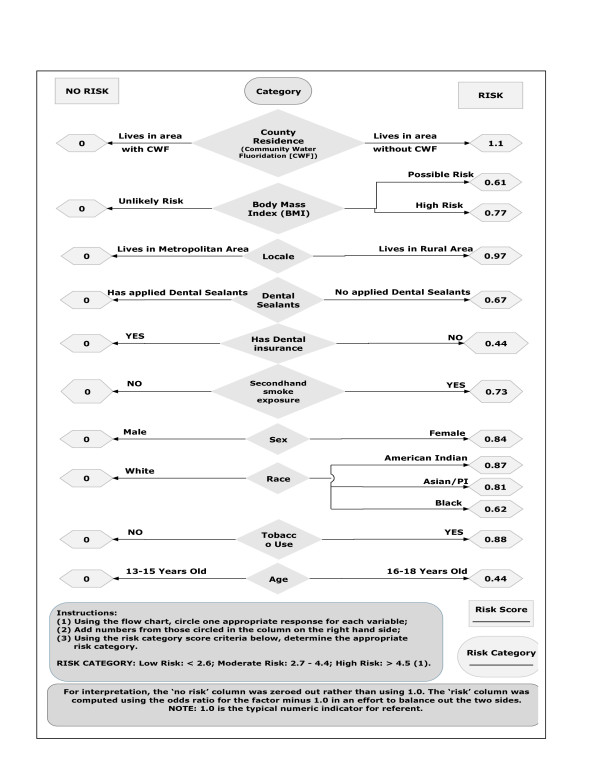
**Theoretical Model for Assessing Dental Caries in Nevada Youth **[10].

After a thorough review of the literature, the only previously established population-based dental caries categories for adolescents were those found in a Policy and Practice bulletin published by the World Health Organization on the Global Burden of Oral Health [1]. Because this is an instrument intended for population-based screenings and not for individual diagnostic purposes, these categories were selected. The only previously established categories for children were established by authors of the WHO's Policy and Practice paper on the Global Burden of Oral Health [1]. As a starting point and in an effort to standardize the DMFT categories these same categories were used as benchmarks (Low: ≤2.6; Moderate: 2.7-4.4; High: ≥4.5) [1,10]. Beta weight comparisons were used in establishing the relative contribution of these variables with no presence of multicollinearity. The multifactorial Risk Factor Model was then developed using odds ratios (Figure 1) [10]. With odds ratios, an odds ratio of 1 implies that the event is equally likely in both the comparison group [referent] and the group being measured, a score greater than one implies that the event is more likely in the group being measured, and a score less than one implies that the event is less likely in the group being measured. For ease of interpretation, the 'no risk' column [on the left side of the flow chart] was zeroed out rather than using 1.0 which is the typical numeric reference indicating the risks are equal between the comparison group [referent] and the group being measured. The 'risk' column [on the right side of the flow chart] indicates the amount of risk for a particular category based on results from the previous study minus 1 to balance out the two sides of the flow chart. That is, both sides were subtracted by 1.0, leaving the left at zero for each factor and the right with the original odds ratio minus 1.0 for each factor. The users simply circle the appropriate response for each factor and upon completion they add the numbers in the 'risk measure' column [odds ratios] in the column on the right hand side. The numbers for each factor are added to create a total 'risk score' and this value is then compared to the scoring criteria categories [1,10].

### Selection of Participants

Researchers examined retrospective data from an oral health surveillance initiative that conducted over 51,000 screenings on children 13 to 18 years of age, attending public/private schools in Nevada across six academic years (2002/2003-2007/2008). The University of Nevada Las Vegas Institutional Review Board approved this study to assure student confidentiality.

The subset of the database was used in the original study [10] which described the development of the mulitfactorial Risk Factor Model in this study. The oral health screening data were collected by trained, calibrated dentists licensed to practice in the State of Nevada [Nevada Statutes and Regulations, Chapter 631, Dentistry and Dental Hygiene]. Inter-rater and intra-rater reliability between the examiners were computed with intraclass correlation coefficients (ICC) (0.81, p < 0.001 and 0.98, p < 0.001 respectively) [11].

### Defining the Standard Used for Population-based Screening

Comparisons between an existing standard and the proposed Risk Factor Model was established to provide sufficient confidence in the instruments ability to distinguish between those with disease from those without; in this case accuracy of the Risk Factor Model in the prediction of future caries risk in Nevada adolescents in a pubic health setting. Any standard used for this purpose would be defined as a single tool (or combination of) tool(s) in support of that outcome [12]. Because this Risk Factor Model was designed to be used in population-based screenings, the gold standard selected for establishing caries prevalence and disease status was the DMFT (decayed, missing, and filled teeth) Index that was developed in 1938 by Klein et al. [13]. Historically, surveys using the Decayed, Missing and Filled Tooth (DMFT) Index have provided data that was translated into future predictions, such as the potential number of teeth to be restored and extracted based on the strength of impact imposed by risk factors identified [14-18]. A review of the literature found that past caries experience was confirmed as the most significant predictor of future caries development, even beyond that of bacterial and socioeconomic factors [16]. The DMFT Index is the criterion still used in large-scale national population-based studies, such as National Health and Nutrition Examination Survey [NHANES] to define caries prevalence and severity. Individuals with a DMFT Index ≥1 are classified as 'with disease' while those with a DMFT Index = 0 are classified 'without disease' [3,13]

An important challenge in screening practices in the public health arena is the ability to correctly identify individuals who do and do not have a disease [19-21]. The screening should be defined as a means for early detection and referral for potential treatment of a disease that is available to a population. This process includes the screening and the follow-up evaluation for those who are considered high risk [19-21]. Characteristics of a successful screening include low cost, minimal risk, convenience, validity, and reliability. Therefore the screening must have a high degree of reliability and validity. This provides a basis for targeting at-risk populations for primary prevention.

Consensus recommendations published by National Institutes of Health stated that individuals with moderate to high risk for dental disease should be identified as early as possible so aggressive strategies could be adopted [9]. Risk Factor scores were calculated for all subjects using the Multifactorial Risk Factor Model parameters [10]. In keeping with NIH criteria for management of caries, operational definitions were created. Individuals with moderate Risk Factor scores (2.7-4.4) and high Risk Factor scores (≥4.5) were collapsed into one category and identified as testing 'positive'. Those with low Risk Factor scores (≤2.6) were considered as testing 'negative'.

### Statistical Analysis

The key parameters used in defining the utility of this Risk Factor Model included sensitivity, specificity, predictive value positive (PVP), predictive value negative (PVN), and disease prevalence [19-22]. When attempting to assess biologic variations within human populations, the tools used should distinguish between those with normal and those with abnormal results and thus allow for a better understanding of how different characteristics are distributed in the population being studied [19]. Therefore a screening tool (Risk Factor Model) is expected to adequately distinguish between those who have the disease [or characteristic] (sensitivity) from those who do not have the disease (or characteristic) (specificity) [19-22].

Screenings were evaluated for feasibility [19-22], with measures that included: predictive value positive (PVP), and predictive value negative (PVN). PVP is defined as the proportion of individuals who tested positive and actually had the disease (or characteristic) at the time screening was conducted. A high PVP implies that the screening program is effective because it detects a large proportion of actual cases among individuals with positive results. PVN is defined as the proportion of individuals who tested negative and were without the disease (or characteristic). It should be noted, that for any population-based screenings, the PVP drops as the prevalence of the disease decreases. Conversely, the PVN rises as the prevalence of the disease decreases. Low prevalence rates generally infer that persons being tested do not have the disease. This dependency on accurate prevalence rates required the use of a representative sample of the population studied and was necessary for calculating predictive values. Direct measures by the licensed dentists in this initiative allowed for sufficient confidence in identification of cases of dental caries. Subsequently, direct measures of prevalence rates (DMFT indices) were computed using actual screening data, thus considered valid for the purposes of identifying dental caries prevalence [3,10].

Pairwise matching of the Multifactorial Risk Factor Model data classifications was used to produce two-by-two tables for illustration of calculation methods according to standard epidemiologic procedures [19-22]. Cronbach's alpha was used to assess the reliability of the measure used across all six years [23]. Table 1 illustrates the classification of data necessary for reliability and validity analyses [19-22]. Cell 'a' holds the number of subjects who tested positive (DMFT ≥ 2.7) and classified 'with disease' (DMFT ≥ 1); Cell 'b' holds subjects who tested positive (DMFT ≥ 2.7) and classified 'without disease' (DMFT = 0); Cell 'c' holds subjects who tested negative (DMFT ≤ 2.6) and classified' with disease' (DMFT≥1); and cell 'd' includes subjects who tested negative (DMFT ≤ 2.6) and classified 'without disease (DMFT = 0). Ideally all tested subjects were expected to fall into 2 cells in the upper left and lower right corner, however, this is rare when conducting population-based screenings [19-22]. In selecting a 'cut-off' level for determining if someone tests positive or negative, or in this case the operational definitions previously identified, was a consideration in developing the multifactorial Risk Factor Model. The choice of a higher or lower cutoff level for screening therefore depended on the potential for obtaining higher false positives and false negatives. Computing sensitivity, specificity, PVP and PVN was necessary in evaluating this Risk Factor Model to address this concern.

**Table 1:** Classification of Data for Reliability and Validity Analyses (Two-by-Two Computation Table)

Assessment Results	With Disease DMFT Score ≥ 1.0	Without Disease DMFT Score = 0	Totals
High Caries Risk [Moderate + High Risk Score]	True Positive [TP] a	False Positive [FP] b	Total test positives a + b

Low Caries Risk [Low Risk Score]	False Negative [FN] c	True Negative [TN] d	Total test negatives c + d

**Totals**	**Total with disease** **a + c**	**Total without disease** **b + d**	**Total population** **a + b + c + d**

**Calculations**			
Specificity a/a+c	Sensitivity d/b+d	Predictive Value Positive a/a+b	Predictive Value Negative d/c+d

Note. a] subjects who tested positive and classified 'with disease'; b] subjects who tested positive and classified 'without disease'; c] subjects who tested negative and classified' with disease'; and d] subjects who tested negative and classified 'without disease

## Results

The disease classification two-by-two table results of subjects' classifications for each academic year were summarized in Table 2. Table 2 describes each year's totals that were used for the computation of specificity, sensitivity, PVP, PVN, and prevalence. Each year was computed individually so that data could be compared across the six years of data collection.

**Table 2:** Disease classification two-by-two table results of subjects' classifications for each academic year

Academic Year	Classifications	With Disease DMFT Score > 1.0	Without Disease DMFT Score = 0	Totals
2002/2003	High Caries Risk	4098	407	**4505**
	
	Low Caries Risk	1366	2304	**3670**
	
	**Totals**	**5464**	**2711**	**8175**

2003/2004	High Caries Risk	6890	729	**7619**
	
	Low Caries Risk	1722	2800	**4522**
	
	**Totals**	**8612**	**3529**	**12,141**

2004/2005	High Caries Risk	3296	586	**3882**
	
	Low Caries Risk	1041	2071	**4522**
	
	**Totals**	**4337**	**2657**	**6994**

2005/2006	High Caries Risk	4963	641	**5604**
	
	Low Caries Risk	1090	2560	**3650**
	
	**Totals**	**6053**	**3201**	**9254**

2006/2007	High Caries Risk	3882	274	**4156**
	
	Low Caries Risk	686	2471	**3157**
	
	**Totals**	**4568**	**2745**	**7313**

2007/2008	High Caries Risk	3842	667	**4509**
	
	Low Caries Risk	1073	1836	**2909**
	
	**Totals**	**4915**	**2503**	**7418**

Table 3 provides a summary table of computations for each academic year for specificity, sensitivity, PVP, PVN, and prevalence. The 95% confidence intervals for each academic year were computed to provide an estimated range of values to account for any unknown population parameters and to further determine the probability that the confidence intervals produced contained the true parameter value [19-22]. The predictive values of the Risk Factor Model across all six academic years for sensitivity ranged from 75% - 85%; specificity ranged from 73% - 90%; PVP ranged from 85-93%; and PVN resulted in a range of 62% - 78%. The overall reliability of the instrument was substantial (r = 0.875, p < 0.001) when measured across all six years of data [23] indicating a stable, reliable predictive capacity of the Risk Factor Model.

**Table 3:** Summary Table of Computations by Academic Year for Specificity, Sensitivity, PVP, PVN, and Prevalence

Year			Predictive Value %		
				
	Sensitivity % [95% CI]	Specificity % [95% CI]	Positive [95% CI]	Negative [95% CI]	Prevalence % [95% CI]
2002/2003	75 [71.04-78.96]	85 [78.80-91.20]	91 [87.61-94.39]	64 [57.73-70.27]	67 [63.63-70.37]
2003/2004	80 [76.04-83.96]	79 [72.80-85.20]	90 [86.61-93.39]	62 [55.73-68.27]	71 [67.63-74.37]
2004/2005	76 [72.04-79.96]	78 [71.80-84.20]	85 [81.61-88.39]	67 [60.73-73.27]	62 [58.63-65.37]
2005/2006	82 [78.04-85.96]	80 [73.80-86.20]	89 [85.61-92.39]	70 [63.73-76.27]	65 [61.63-68.37]
2006/2007	85 [81.04-88.96]	90 [83.80-96.20]	93 [89.61-86.39]	78 [71.73-84.27]	63 [569.63-66.37]
2007/2008	78 [74.04-81.96]	73 [66.80-79.20]	85 [81.61-88.39]	63 [56.73-69.27]	66 [62.63-69.37]

Note. 95% CI was computed using t-distribution.

## Discussion

The purpose of this study was to determine whether the Risk Factor Model previously developed was a valid instrument that could be used by non-dental professionals in public health settings for predicting future caries risk among Nevada adolescents and that could lead to appropriate prevention and referral practices. In practical terms, the predictive capacity of the Risk Factor Model was not very different across the six years. Differences in yearly variability can be attributed to two factors: 1) differences in the prevalence rates from year-to-year, and 2) high reported prevalence rates. Subjects' overall mean caries prevalence across all six academic years was 66% with a range reported from 62% in year 3 (2004/2005) to 71% in year 2 (2003/2004). These differences could have resulted in the variability found in sensitivity, specificity, PVP and PVN from year-to-year [24]. Differences in these predictive values could also have been attributed to the sample's high caries prevalence rates. This study confirmed that dental caries remained a common chronic disease among Nevada youth during the years studied, presenting with a higher prevalence rate than the national average (66% vs. 59%) for adolescents and higher than the Healthy People 2010 goal for Nevada of 51% [3,25]. This study confirmed average prevalence rates (66%) were representative of the Nevada adolescent population reporting 61% prevalence in 2006 [26].

Although direct measures were obtained by licensed dental professionals, self-report information of demographic variables in the initial oral health screening warrants some caution in interpreting data. Data collection and screening protocols were documented extensively with quality control guidelines in place. Due to student confidentiality issues, students were not tracked over time; however collection of cross-sectional data of Nevada youth from all demographics across all six years helped strengthen interpretations and provided a strong representation of the Nevada adolescent population.

The cut-off values used in this study were adopted from a previous study conducted in the same population [10]. It should be noted that cut-off values for the various categories (low, moderate, and high risk) could vary in different populations. For instance in some populations 90% of subjects may be low risk if a DMFT <2.6 is used while in other populations only 10% may fall in that category. Therefore, caution should be used when generalizing to different populations.

### Conclusion and Clinical Relevance of Findings

These results indicated that this Risk Factor Model could be used to predict future caries risk with sufficient confidence in conjunction with other risk assessments by non-dental health practitioners, such as school nurses/nurse practitioners, health educators, and physicians in population-based settings. Interpretation of epidemiological data by investigators is generally considered subjective. A review of the literature showed a wide range of acceptable levels regarding sensitivity, specificity, predictive value positive, and predictive value negative [12,26-29]. Ranges from 70% to 100% have been considered good to very good or valid levels. Defining validity of an instrument has always required that many factors be considered. Standards to assess the validity of tests used by dental professionals for diagnostic purposes must be set higher than standards set forth in population-based screenings used by non-dental health practitioners to guide them in assessing potential future caries risk for use in prevention and referral practices. Thus, such a Risk Factor Model should be looked at from the 'practicality' aspect for its intended purpose.

Previous studies of school-based dental screening have found it to stimulate follow-up for dental services of those socially deprived [30]. What must be considered is the effectiveness of population-based screenings of the number identified as high risk who subsequently go on to receive appropriate treatment. There is some evidence to suggest that children who are identified for follow-up services after school-based dental screenings leads to improved attendance with dental professional [30].

## Conclusion

Because non-dental healthcare professionals (e.g., such as school nurses/nurse practitioners, health educators, and physicians, etc.) have frequent contact with adolescents and their parents/guardians, during health screenings at school or in the community, a valid Risk Factor Model is an excellent tool that can be used in conjunction with other risk assessments to help guide early detection, prevention, and referral practices. Results indicated that prediction success was relatively high, and the Risk Factor Model was found to have satisfactory predictive power. Thus, this study provided sufficient confidence in the Risk Factor Model as a population-based screening measure and indicates that the Risk Factor Model may be useful in public health practice as a reliable scale for predicting future caries risk for early detection and referral practices by non-dental health practitioners. Future prospective studies designed to examine the practical use of the Risk Factor Model by Nevada non-dental healthcare professionals could further support these findings.

## Competing interests

The authors declare that they have no competing interests.

## Authors' contributions

MD, GD, KH, CM, DC have been engaged in the multi-year development of the surveillance program, including developing the protocols and guidance for examiners, data collection as well as ongoing quality control. MD performed the statistical analysis. The paper was drafted by MD; GD, KH, CM, and DC contributed to its completion. All authors have read and approved the final manuscript.

## Pre-publication history

The pre-publication history for this paper can be accessed here:

http://www.biomedcentral.com/1472-6831/11/18/prepub
